# Resource supply governs the apparent temperature dependence of animal production in stream ecosystems

**DOI:** 10.1111/ele.13608

**Published:** 2020-10-01

**Authors:** James R. Junker, Wyatt F. Cross, Jonathan P. Benstead, Alexander D. Huryn, James M. Hood, Daniel Nelson, Gísli M. Gíslason, Jón S. Ólafsson

**Affiliations:** ^1^ Department of Ecology Montana State University Bozeman MT 59717 USA; ^2^ Department of Biological Sciences University of Alabama Tuscaloosa AL 35487 USA; ^3^ Department of Evolution, Ecology, and Organismal Biology The Ohio State University Translational Data Analytics Institute The Aquatic Ecology Laboratory Columbus OH 43212 USA; ^4^ Department of Biology University of Oklahoma Norman OK 73019 USA; ^5^ University of Iceland Institute of Life and Environmental Sciences Reykjavík Iceland; ^6^ Institute of Marine and Freshwater Fisheries Reykjavík Iceland; ^7^ Present address: Louisiana Universities Marine Consortium Chauvin LA 8124 LA‐56 USA

**Keywords:** climate change, light, metabolic theory, seasonality, secondary production, temperature

## Abstract

Rising global temperatures are changing how energy and materials move through ecosystems, with potential consequences for the role of animals in these processes. We tested a central prediction of the metabolic scaling framework—the temperature independence of animal community production—using a series of geothermally heated streams and a comprehensive empirical analysis. We show that the apparent temperature sensitivity of animal production was consistent with theory for individuals (*E*p_ind_ = 0.64 vs. 0.65 eV), but strongly amplified relative to theoretical expectations for communities, both among (*E*p_among_ = 0.67 vs. 0 eV) and within (*E*p_within_ = 1.52 vs. 0 eV) streams. After accounting for spatial and temporal variation in resources, we show that the apparent positive effect of temperature was driven by resource supply, providing strong empirical support for the temperature *independence* of invertebrate production and the necessary inclusion of resources in metabolic scaling efforts.

## Graphical abstract

We tested a central prediction of the metabolic scaling framework—the temperature independence of animal community production—using a series of geothermally heated streams and a comprehensive empirical analysis. After accounting for spatial and temporal variation in resources, we show that the apparent positive effect of temperature was driven by resource supply, providing strong empirical support for the temperature independence of invertebrate production and the necessary inclusion of resources in metabolic scaling efforts.

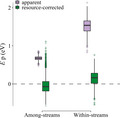

## INTRODUCTION

Increasing temperatures influence patterns and processes in Earth’s ecosystems with potentially large consequences for the provision of ecosystem goods and services (Montoya and Raffaelli, 2010; IPCC 2018). Many important ecosystem services (e.g. food and fibre production) are closely tied to fluxes of energy and materials through ecological networks (Raffaelli *et al*., 2002). Although significant research has focused on how warming influences the role of heterotrophic microbes in energy and material cycling (Allison *et al*., 2010; Song *et al*., 2018), less effort has been directed towards assessing the role of animals, perhaps due to the perception that their influence at the ecosystem level is less significant. A growing literature, however, demonstrates strong effects of animals on ecosystems (Carpenter *et al*., 1985; Wallace and Webster, 1996; Schmitz *et al*., 2018), underscoring the need to understand how warming influences energy and material flux through animal communities (Petchey *et al*., 1999; Brose *et al*., 2012).

An effective approach for linking animal communities to ecosystem processes is the quantification of secondary production (Waters, 1969; Waters, 1977; Benke and Huryn, 2010). Broadly, secondary production, or the formation of heterotrophic biomass over time, is an ecosystem flux that incorporates individual and population‐level characteristics (e.g. individual size and growth, biomass, reproduction, survivorship), many of which are influenced by temperature (Benke, 1993; Brown *et al*., 2004). Thus, secondary production provides an integrative measure ideally suited for understanding the influence of warming on animals and their role in ecosystems.

The metabolic theory of ecology (MTE) provides a useful framework for understanding the influence of temperature on animal metabolism and secondary production. This theory focuses on two fundamental drivers of individual metabolic rate—body size and temperature (Brown *et al*., 2004). The effects of body size, *M_i_* (mass), and temperature, *T* (Kelvin), on individual metabolic rates can be described by the equation:

(1) $$I\;=\;i_{0}M_{i}^{0.75}e^{‐E/kT}$$

where, *I* is individual metabolic rate, *i_0_* is a normalisation constant, *E* represents the mean activation energy of cellular respiration (0.60–0.70 eV) and *k* is the Boltzmann constant (8.62 10^−5^ eV K^‐1^; Gillooly *et al*., 2001; Brown *et al*., 2004). This equation, which describes the metabolic energy demand of an organism, including energy required for maintenance, growth and reproduction may be extended to make predictions about secondary production at higher levels of organisation.

To move from individual‐ to population‐level predictions about secondary production, we must consider two factors, the product of which is secondary production: population biomass (*B*) and biomass turnover rate (production to biomass relationship; *P:B*), as well as how these factors are influenced by body size and temperature (Cross *et al*., 2015). With respect to population *B* (mass area^−2^), MTE predicts:

(2) $$B\;\approx \;\left[R\right]\;M^{0.25}e^{E/kT}$$

where *[R]* represents a generic term for resource supply. For a given resource supply, this equation predicts that total population biomass should increase with average body size (i.e. *M* ^0.25^) and decrease with temperature at an exponent approximating the activation energy of cellular respiration (i.e. *E* = 0.60–0.70 eV; Savage, 2004).

Population biomass turnover rate (*P:B*; time^−1^), is predicted by MTE as follows:

(3) $$P:B\;\approx \;M^{‐0.25}e^{‐E/kT}$$

where *P:B* is expected to decline with body size with a negative, but similar exponent (i.e. −0.25), and increase with temperature with a positive, but similar exponent to that of *B* (i.e. 0.6–0.7; Brown *et al*., 2004; Huryn and Benke, 2007). Because secondary production (*P*; mass area^−2^ time^−1^) is the product of *B* and *P:B*, the effects of body size and temperature cancel and population‐level production is controlled only by resources supplied to the population (i.e. *P ≈ [R]*; Damuth, 1987; Allen *et al*., 2002; Huryn and Benke, 2007; Cross *et al*., 2015).

Although rarely considered explicitly, knowledge about resource supply, and how it varies with temperature, is central to predicting responses of secondary production at the population and community levels. Indeed, predicting the apparent temperature dependence of population‐level production is only possible if resource use by individual populations can be accurately quantified. Thus, predictions outlined above may be more appropriately applied to trophic groups or communities feeding on a common, quantifiable resource pool because any decrease in resources used by a given population may be offset by elevated resource use by others (Van Valen, 1973; White *et al*., 2004). Furthermore, resource supply is rarely distributed uniformly in time and typically covaries with changes in light, nutrient availability and temperature. Because of this covariation, disentangling the role of resource supply versus temperature in controlling consumer metabolism, biomass and secondary production remains an important pursuit, especially in the context of global change.

Here, we quantified the apparent temperature dependence of invertebrate production and its individual components – *B* and *P:B* – from the individual to the community along natural stream temperature gradients among and within streams (annual mean temperature range: 5–28 °C). Previous research in these streams has shown a strong positive effect of temperature on primary production, both among streams of different temperatures (Demars *et al*., 2011; Padfield *et al*., 2017) and within streams across seasons (O'Gorman *et al*., 2012; Hood *et al*., 2018). Thus, predictions based on MTE must account for spatial and temporal variation in the resource base. At the individual‐level, we expected that mass‐specific growth rates would follow theoretical expectations for metabolic rates (Brown *et al*., 2004), given differences in body size and temperature. We had no *a priori* prediction about secondary production at the population‐level, given the likely large interspecific differences in resource use. At the community‐level, we expected secondary production to vary strongly over space and time, driven by (1) variation in resource supply among streams and (2) seasonal variation in resources within each stream. By correcting for this spatial and temporal variation in resources, we show that apparent positive effects of temperature on production are driven by resource supply, providing strong empirical support for the theoretical temperature *independence* of animal production.

## MATERIAL AND METHODS

We studied six streams within the Hengill geothermal field of southwestern Iceland (64°03´N 021°18´W) that varied in mean annual temperature. The Hengill region is characterised by indirect geothermal heating of groundwater (Árnason *et al*., 1969), leading to heterogeneity in water temperatures (4.5–54.0 °C), but similar solute chemistries (Friberg *et al*., 2009). These conditions create a natural laboratory for isolating the effects of temperature on ecosystem processes (Hannesdóttir *et al*., 2013; O'Gorman *et al*., 2014; Nelson *et al*., 2017b). We selected streams to maximize the temperature range, w minimising differences in structural aspects of primary producers (i.e. minimal biomass of large macrophytes). In each stream, we measured temperature and water depth every 15 min from July 2010 through August 2012 (U20‐001‐01 water‐level logger, Onset Computer Corp. Pocasset, MA, USA). Temperature time‐series were adjusted from Celsius to Kelvin to standardised inverse Boltzmann temperature centred on 15°C (1/[*kT*_15_ *– kT*]). Light availability in the watershed was measured every 15 min from atmospheric stations (HOBO pendant temperature/light UA‐002‐64, Onset Computer Corp. Pocasset, MA, USA).

### Invertebrate sampling

We sampled macroinvertebrate communities approximately monthly from July 2011 to August 2012 in four streams and from October 2010 to October 2011 in two streams used in a previous study (*n* = 6 streams). The two streams were part of an experiment beginning in October 2011, therefore overlapping years were not used to exclude the impact of experimental manipulations (see Nelson *et al*., 2017a,b). Inter‐annual comparisons of primary and secondary production in previous studies showed minimal differences among years in unmanipulated streams, suggesting that combining data from different years would not significantly bias our results (Nelson *et al*., 2017b; Hood *et al*., 2018). We collected five Surber samples (0.023 m^2^, 250‐µm mesh) from randomly selected locations within each stream. Following placement of the sampler, inorganic substrates were disturbed to a depth of *c*. 10 cm and invertebrates and organic matter were dislodged from stones with a brush. Samples were then preserved with 5% formaldehyde. In the laboratory, we split samples into coarse (>1 mm) and fine (<1 mm but > 250 µm) fractions using nested sieves and removed invertebrates from each fraction under a dissecting microscope (10–15 × magnification). For particularly large samples, fine fractions were sub‐sampled (1/2–1/16) using a modified Folsom plankton splitter and invertebrates were removed from subsamples. Subsamples were scaled to the rest of the sample by assuming a similar abundance and body‐size distribution. Invertebrates were identified to the lowest practical taxonomic level (usually genus) using relevant sources (Peterson, 1977; Merritt *et al*., 2008; Andersen *et al*., 2013). Body lengths of individuals were measured to the nearest 0.25 mm and body size was estimated using length–mass regressions (Benke *et al*., 1999; O'Gorman *et al*., 2012; Hannesdóttir *et al*., 2013). Taxon‐specific abundance and biomass were scaled to m^−2^.

### Individual growth

Growth rates of macroinvertebrates were estimated empirically using taxon‐appropriate methods. First, for common taxa (i.e. *Radix balthica*, *Simulium* spp. and dominant Chironomidae species), live individuals (*n* = 5–15) were collected in the field, separated into small size categories (*c*. 1 mm range in length) and photographed next to a field micrometer. Individuals were then placed into the stream within pre‐conditioned (> 7 days) clear PVC tube chambers with mesh ends (0.25–0.5 mm depending on taxon). Chambers were removed after 7‐15 days and individuals were photographed as above. Body lengths were measured from initial and final photographs using image analysis software (ImageJ; Schindelin *et al*., 2012) and converted to mass (mg ash‐free dry mass [AFDM]) using length‐mass regressions. Instantaneous growth rates (*g*, d^−1^) were estimated as changes in mean body size divided by the time between initial and final measurements as:

(4) $$g\;=\;\log_{e}\left(W_{t+\Delta t}/W_{t}\right)/\Delta t$$

where, *W* represents individual mass at some time, *t*, and Δ*t* is measured in days.

Secondly, for taxa with individuals that develop synchronously, and therefore had visually distinguishable cohorts, we examined temporal changes in size‐frequency distributions and calculated growth rates and uncertainty using a bootstrap technique similar to that described in Benke and Huryn (2017). Size‐frequency histograms were visually inspected for directional changes in body‐size distributions. For each date, size‐frequency distributions were resampled with replacement (*n* = 500) and growth rates were estimated using Equation 4 to create vectors of taxon‐, size‐ and date‐specific growth rates. We prevented calculation of negative growth rates by requiring that resampled *W*_t+Δt_ > *W*_t_. To estimate growth rates of additional taxa for which we could not measure growth, we developed stream‐specific growth equations using the empirically derived growth estimates from chambers and size‐frequency distributions described above. From these measurements, we also estimated the general mass‐ (*a*) and temperature‐dependence (*E*p_ind_) of individual growth (*g_ind_*) by multivariate regression of a linearised modification of eqn 1: log_e_(*g_ind_*) = a * log_e_(*M_i_*) + Ep_ind_ * 1/kT.

### Population‐ and community‐level secondary production

Secondary production of populations was estimated using the instantaneous growth method (Benke and Huryn, 2017). Briefly, this method calculates secondary production as follows:

(5) $$P\;=\;g\;\Delta t\left(\left(B_{t}+\;B_{t+\Delta t}\right)/2\right)$$

where, *P* is secondary production, *g* is mass‐specific growth, *Δt* is days between sampling dates and *B_t_* is population biomass at time *t*. To estimate uncertainty, we used a bootstrap technique that resampled instantaneous growth rates derived above, as well as abundance and size distributions from individual samples. For each of 1000 iterations, growth rates were multiplied by mean interval biomass for each size‐class and *Δt* to estimate total size‐class production for each sampling interval. Size‐classes were summed to calculate total population‐level production for each interval. Within each interval, bootstrapped vectors were summed across populations to estimate community‐level production. Interval secondary production was standardised to a daily timescale by dividing by the number of days between sampling events (mg AFDM m^−2^ d^−1^). For annual estimates, population and community production were summed across all time intervals.

### Temperature and body‐size scaling of community secondary production, B and P:B

The apparent temperature dependence (Anderson‐Teixeira *et al*., 2008) of annual secondary production among streams (*E*p_among_) was estimated by bootstrapped ordinary least squares (OLS) regression of log*_e_*‐transformed annual secondary production against mean annual standardised Boltzmann‐temperature. To estimate the apparent temperature dependence of annual community *B* (*E*b_among_) and *P:B* (*E*pb_among_), we corrected for body‐size differences among communities by first calculating the annual mean body size of each taxon for each of the 1000 bootstrapped estimates. We then derived 1000 estimates of the mean body size of the community, weighted by the biomass of individual taxa (Yvon‐Durocher and Allen, 2012; Barneche *et al*., 2014). Vectors of bootstrapped invertebrate community *B* or *P:B* were then multiplied by the weighted community body‐size raised to the assumed body‐size scaling exponents in Equations 2 and 3 (i.e. ± 0.25; Yvon‐Durocher and Allen, 2012). While the value of these exponents is subject to debate (Cyr and Walker, 2004), we applied them to capture a central tendency based on diverse taxa. Corrected *B* and *P:B* were then log*_e_*‐transformed and regressed against annual mean standardised Boltzmann‐temperature to estimate *E*b_among_ and *E*pb_among_ among streams.

We used bootstrapping to estimate uncertainty in apparent temperature dependences (i.e. *E*p, *E*b and *E*pb). We resampled with replacement our bootstrapped estimates of secondary production, mass‐corrected *B* and mass‐corrected *P:B* in each stream, and calculated the OLS slope coefficient between these values and temperature. We repeated this procedure 10 000 times to create a distribution of slope coefficient estimates. Confidence bounds of the temperature dependences were calculated as the 2.5% and 97.5% percentile of bootstrapped slope estimates. We used a similar approach to estimate the apparent temperature dependence of secondary production within streams over the course of a year using mean sampling interval temperature (*E*p_within_; i.e. intra‐annual slopes).

### Resource supply and the temperature dependence of secondary production

#### Among streams

To estimate variation in resource supply among streams, we quantified biofilm chlorophyll *a* on each sampling date (*n* = 10–12) from five random stones in each stream. The area of a 35‐mm slide mount (8.05 cm^2^) was scrubbed from each stone with a wire brush and rinsed into a plastic amber bottle. Subsamples of the biofilm slurry were filtered onto glass‐fibre filters (Whatman GF/F; 0.7‐μm pore size), extracted overnight with acetone, analysed fluorometrically for chlorophyll *a* content (Turner Designs, Sunnyvale, CA USA) and standardised to mg m^−2^. Distributions of mean annual chlorophyll *a* biomass were constructed by resampling monthly samples with replacement and calculating annual means. The apparent temperature dependence of annual chlorophyll *a* (*E*b_chla_ ± 95% CI) was estimated through 10 000 bootstrapped OLS regressions of chlorophyll *a* vs. mean annual standardised Boltzmann temperature. Although chlorophyll *a* biomass represents only a portion of total resource supply and is a pool instead of a flux like production, previous research at our study site has shown that (1) macroinvertebrate diets are dominated by components of biofilms (e.g. diatoms, green algae; O'Gorman *et al*., 2012; Junker, 2019; Nelson *et al*., 2020) and (2) variation in chlorophyll *a* biomass is correlated with interstream variation in primary production (Padfield *et al*., 2017). Thus, chlorophyll *a* biomass averaged across the year is a reliable proxy for differences in resource supply among streams.

We examined the influences of temperature and resources on secondary production among streams using Akaike’s Information Criterion to assess support for competing models (AICc; Burnham and Anderson, 2002). We assessed multiple null and sub‐models of the full model:

(6) $$\log_{e}\left(P_{\mathrm{among}}\right)\;=\;temp*\;\log_{e}\left(\mathrm{chla}\right)$$

where *P_among_* represents annual community secondary production, *temp* is mean annual standardised Boltzmann temperature and *chla* is mean annual chlorophyll *a* biomass. To test the robustness of model selection, we repeated the model selection exercise 1000 times on resampled estimates of annual secondary production and chlorophyll *a* biomass. The model most frequently showing the lowest AICc score was refit with OLS regression 1000 times on resampled data, and 95% confidence bounds were estimated. We then calculated the apparent temperature dependence as the *temp* coefficient in the temperature‐only model and the resource‐corrected temperature dependence as the *temp* coefficient in the chlorophyll *a*‐temperature additive model.

#### Within streams

Static measures of chlorophyll *a* biomass are less appropriate as a proxy for resource supply within streams because of times during the year when correlations between chlorophyll *a* biomass and primary production are weakened by large variation in production per unit chlorophyll *a* biomass (e.g. low light conditions; Rhee and Gotham, 1981). Thus, we used a tiered approach to examine the influence of temperature and resources on within‐stream secondary production. First, we used an information‐theoretic approach as above to determine the best model for within‐stream secondary production. We constructed a linear mixed‐effects model that included chlorophyll *a* biomass, as well as obvious drivers of resource supply (i.e. light and temperature):

(7) $$\log_{e}\left(P_{\mathrm{within}}\right)\;=\;temp*\log_{e}\left(\mathrm{chla}\right)*\log_{e}(light)+\varepsilon^{s}$$

where *P_within_* is daily community secondary production, and fixed effects included sampling interval means of the standardised Boltzmann temperature (*temp*), chlorophyll *a* biomass (*chla*) and daily light intensity (*light*). In all models, stream identity was treated as a random effect allowing for stream‐specific intercepts (ε*^s^*). We fit null and sub‐models of the fixed effects using maximum‐likelihood estimation with the ‘lme4’ package (Bates *et al*., 2015) and selected the best model based on AICc values. We repeated this process 1000 times on resampled secondary production and chlorophyll *a* data to assess the sensitivity of model selection to uncertainty in these variables. We then refit the model with the highest occurrence of support 1000 times using restricted maximum‐likelihood (REML) on resampled data. We estimated the apparent temperature dependence of within‐stream secondary production (*E*p_within_) as the fixed‐effect coefficient of temperature and calculated the mean and 95% confidence bounds as described earlier.

Finally, to correct for the effect of resource supply on *E*p_within_, we estimated the within‐stream temperature dependence of primary production. First, we constructed a statistical model of gross primary production (GPP) using monthly chlorophyll *a*, light, and temperature data from two study streams (Hood *et al*., 2018; see Appendix S2 Table S4 and Figures S6 and S7 in Supporting Information). The temperature coefficient from this model is an estimate of the within‐stream temperature dependence of GPP (*E*gpp_within_). The resource‐corrected temperature dependency of secondary production within streams was calculated as the difference between *E*p_within_ and *E*gpp_within_. We resampled these distributions and calculated variability and 95% confidence bounds in resource‐corrected values as described earlier. All statistical analyses were conducted in R (R Core Team, 2016).

## RESULTS

Mean annual stream temperatures ranged from 5.0 to 27.2 °C and daily mean temperature showed moderate covariation with light (Pearson’s *r* = 0.67 to −0.09, mean *r = *0.42; Appendix S1, Figure S1 and Table S1).

### Individual growth

Instantaneous growth rates were variable, but generally followed theoretical expectations with body size and temperature (multivariate regression *r* ^2^ = 0.18; Figure S2). Estimates of scaling coefficients for body size (*a*) and temperature (*E*p_ind_) overlapped values predicted by the MTE (*M* ^a^ = −0.18 [95% CI: −0.25 to −0.12] vs. −0.25 and *E*p_ind_ = 0.64 eV [95% CI: 0.47–0.82] vs 0.65 predicted).

### Among‐stream variation in community secondary production

**Figure 1:**
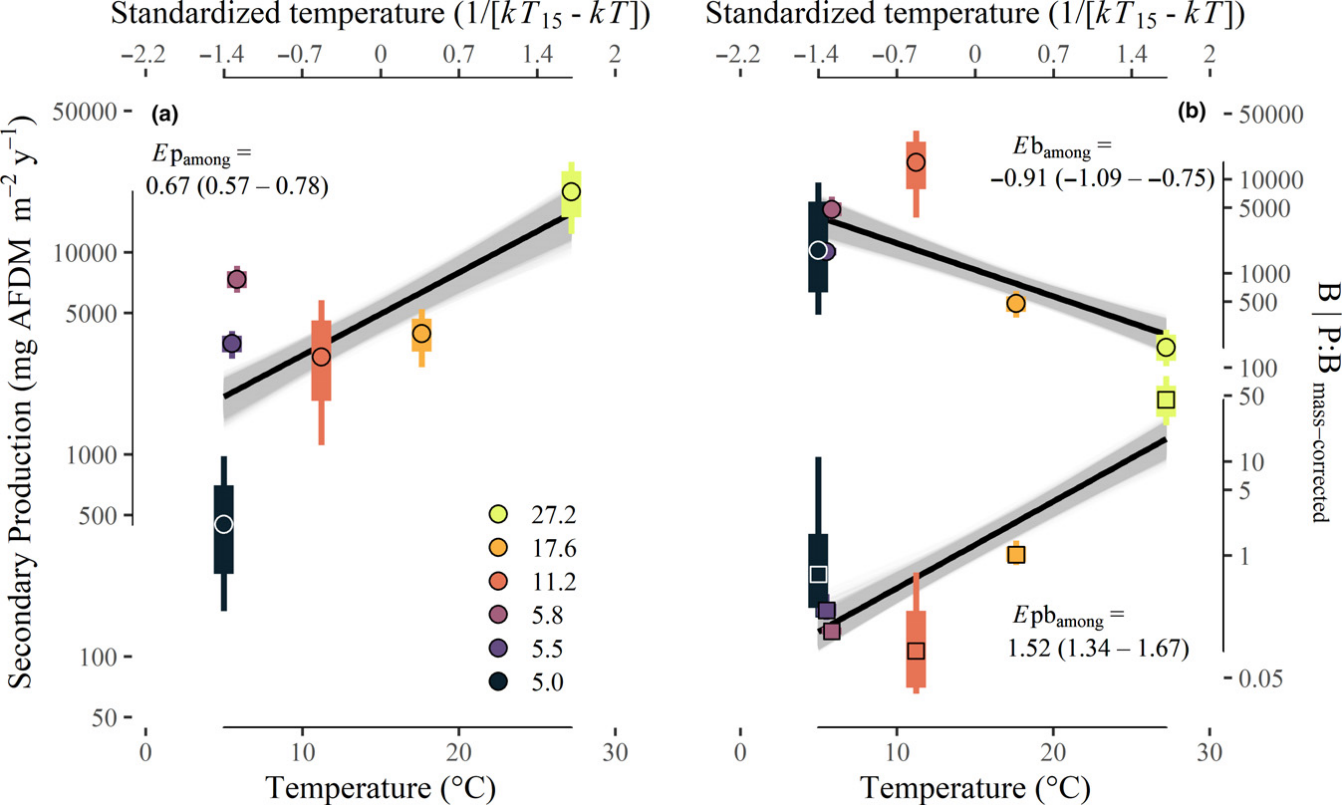
Temperature scaling of annual community secondary production (panel a), annual mass‐corrected community biomass (*B*; mg AFDM m^−2^; panel b; outlined circles and top line) and production:biomass relationships (*P:B*; y^−1^; panel b, outlined squares and bottom line). The mean annual measurement within each stream is represented by points, the 95% quantiles are represented by the bottom and top of each box, and whiskers represent minimum and maximum bootstrapped estimates. The mean (black line) and all bootstrapped linear regressions (grey lines) of the apparent temperature dependence of secondary production (*E*p_among_) are also shown.

Annual production varied widely among populations (Figure S3), and total community secondary production was positively associated with mean annual temperature, increasing *c*. 45‐fold between the coldest and warmest streams (range: 0.45–19.9 g AFDM m^−2^ y^−1^; Fig. 1a). The apparent temperature dependence of community production among streams (*E*p_among_) was 0.67 eV (95% CI: 0.57–0.78), corresponding to a *c*. 9% (95% CI: 8.4–11.4%) increase in production for each 1 °C increase in temperature. The temperature dependence of body size‐corrected *B* and *P:B* exhibited consistent signs (i.e. negative and positive respectively), but steeper temperature dependence than predicted by MTE: *E*b_among_ = −0.91 eV (95% CI: −1.09 to −0.74) vs. −0.65 and *E*pb_among_ = 1.52 eV (95% CI: 1.34–1.67) vs. 0.65 (Table 1; Fig. 1b).

**Table 1:** Apparent temperature dependence (mean estimate and 95% confidence bounds) of secondary production (*E*p), biomass (*B*; *E*b) and production:biomass (*P:B*; *E*pb) relationships among and within streams

	Among streams		Within streams	
	*E*_among_ (95% CI)	*r* ^2^	*E*_within_ (95% CI)	*r* ^2^
Secondary production	0.67 (0.57–0.78)	0.44	–	–
hver (27.2°C)	–	–	1.7 (1.3–2.1)	0.78
st6 (17.6°C)	–	–	0.8 (0.5–1.1)	0.07
st9 (11.2°C)	–	–	3.9 (3.1–4.5)	0.82
st7 (5.8°C)	–	–	4.1 (3.7–4.5)	0.79
oh2 (5.5°C)	–	–	2.7 (2.5–2.8)	0.61
st14 (5.0°C)	–	–	1.7 (0.9–2.3)	0.49
*Biomass_mass‐corrected_*	−0.91 (−1.09 to −0.74)	0.48	–	–
hver	–	–	2.9 (2.5–3.3)	0.78
st6	–	–	0.6 (−0.1–1.4)	0.05
st9	–	–	2.2 (0.0–4.6)	0.24
st7	–	–	2.6 (1.0–4.0)	0.27
oh2	–	–	0.9 (0.6–1.2)	0.09
st14	–	–	1.6 (0.0–2.9)	0.13
*P:B_mass‐corrected_*	1.52 (1.34–1.67)	0.70	–	–
hver	–	–	−1.7 (−2.2–−1.2)	0.37
st6	–	–	0.1 (−0.7–0.7)	0.04
st9	–	–	1.0 (−1.1–3.1)	0.12
st7	–	–	0.3 (−1.0–1.9)	0.04
oh2	–	–	1.8 (1.4–2.1)	0.24
st14	–	–	−1.4 (−2.8 to −0.0)	0.09

Mean annual temperatures (°C) are shown in parentheses following the stream names (e.g. ‘hver’). Estimated apparent activation energy (*E*) and coefficients of determination (*r^2^*) represent the mean estimates derived from the relationship between log*_e_*‐transformed variables and standardised Boltzmann‐temperature (1/[*kT*_15_ *– kT*]). Community *B* and *P:B* were mass‐corrected by biomass‐weighted body size of the community.

### Within‐stream variation in community secondary production

**Figure 2:**
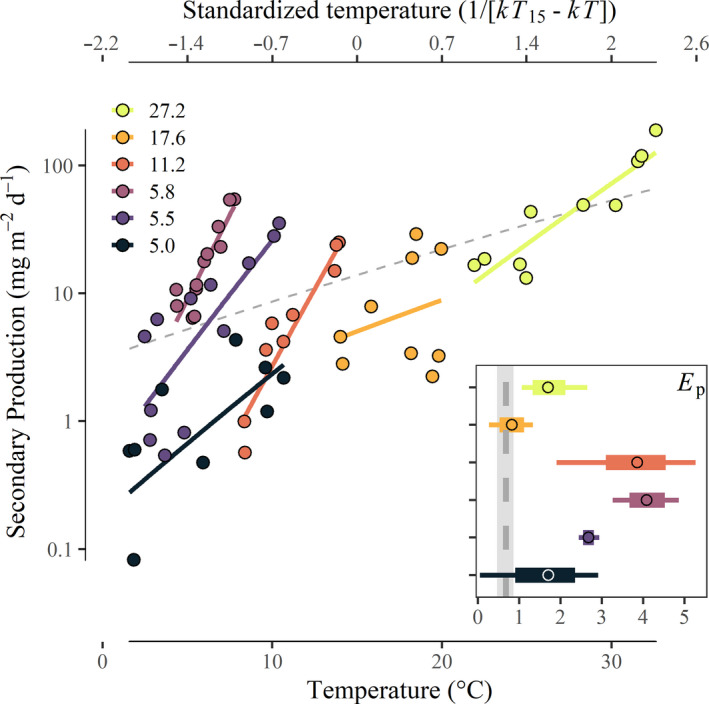
The within‐stream temperature dependence of seasonal community secondary production (solid lines) calculated from mean estimates of secondary production between sampling intervals in each stream (filled points). The inset shows that most streams exhibited steeper temperature dependence of secondary production (*E*p_within_; points represent mean estimates, 95% quantiles are represented by boxes and minima and maxima are represented by whiskers) than across streams at the annual scale (*E*p_among_; dotted line with minima and maximum estimates shaded; adjusted to daily timescale).

Within streams, we observed wide temporal variation in community secondary production (Fig. 2; Figure S4). Minimum daily production ranged from 0.08 to 13.2 mg AFDM m^‐2^ d^‐1^, whereas peak daily production ranged from 4.3 to 188.6 mg AFDM m^−2^ d^−1^. The relationship between temperature and community secondary production within streams was generally much steeper than the relationship among streams (Fig. 2; coloured lines vs. dashed line). The apparent temperature dependence of within‐stream secondary production (*E*p_within_) ranged from 0.8 eV (95% CI: 0.5–1.1; mean temperature: 17.6 °C) to 4.1 eV (95% CI: 3.7–4.5; mean temperature: 5.8 °C; Fig. 2, Table 1).

**Figure 3:**
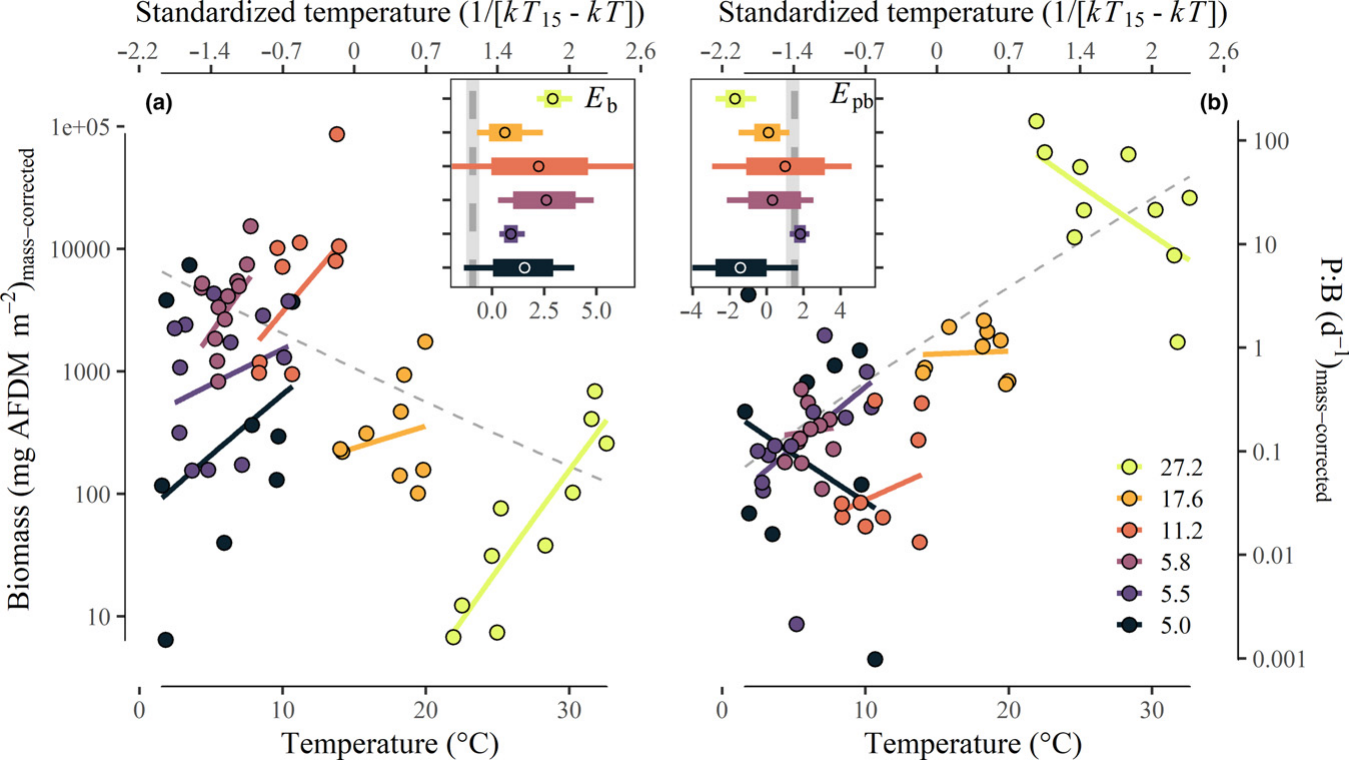
Seasonal relationships between stream temperature and community biomass (panel a) and production:biomass relationships (panel b) corrected for the average community body size. The annual relationship (*E*b_among_ and *E*pb_among_) among streams is shown with dashed grey lines. The estimated relationships within streams are represented by solid coloured lines. The insets show the temperature‐dependence of community *B* (*E*b_within_) and *P:B* (*E*pb_within_) within each stream. Vertical dashed grey lines show the estimated annual temperature dependences among streams.

Seasonal patterns of community *B* (*E*b_within_) and *P:B* (*E*pb_within_) suggested that variation in *B* was much more important than *P:B* in explaining the temperature dependence of community secondary production (Fig. 3). Body size‐corrected *B* was positively related to temperature within all streams (Fig. 3a, coloured lines), in contrast to the negative relationship among streams (Fig. 3a, dashed line) and the negative theoretical expectation. *E*b_within_ ranged from 0.6 eV to 2.9 eV (mean ± 1 SD: 1.8 ± 0.9; Fig. 3a, Table 1). The relationship between *E*pb_within_ and temperature was variable in direction and magnitude, ranging from −1.7 eV to 1.8 eV (mean ± 1 SD: 0.02 ± 1.36; Fig. 3b; Table 1).

### Accounting for resource variation among streams

**Figure 4:**
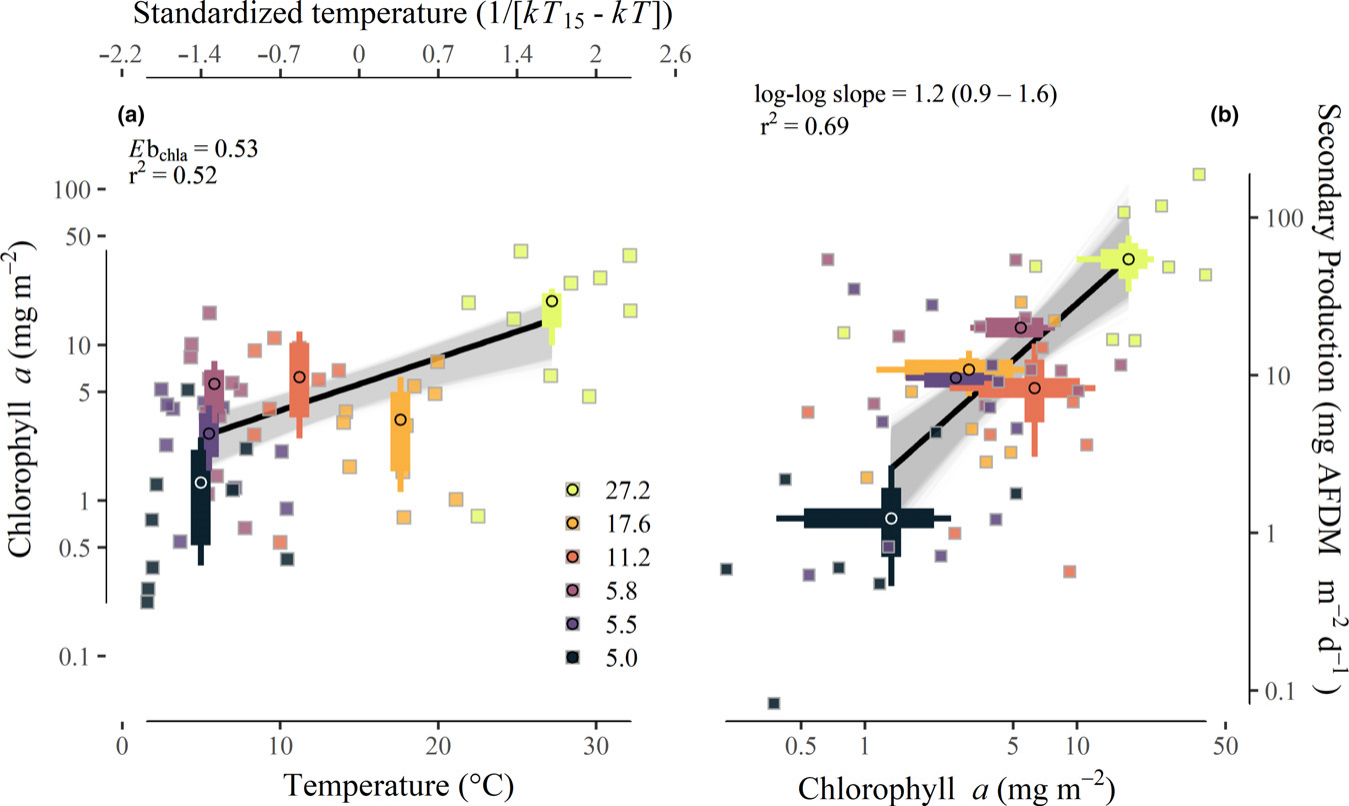
(panel a) Relationship between stream temperature (°C) and chlorophyll *a* biomass. Chlorophyll *a* biomass was positively associated with mean annual temperature (*r^2^* = 0.52) exhibiting a temperature dependence (*E*b_chla_) of 0.53 eV (mean estimate, black line; all bootstrapped regressions, grey lines). Round points represent mean annual values for each stream, boxes represent the 95% quantiles, whiskers show the minima and maxima. Small square points represent means from each sampling interval. (panel b) Annual chlorophyll *a* biomass was positively related to annual secondary production across streams (*r* ^2^ = 0.69; mean, black lines; all bootstrapped regressions, grey lines). Annual production was scaled to daily values by dividing by 365 to allow for visual comparisons with seasonal secondary production estimates. Within streams (small square points), chlorophyll *a* biomass was not associated with secondary production.

Epilithic chlorophyll *a* biomass varied *c*. 180‐fold within and among streams. Mean annual chlorophyll *a* biomass was positively associated with mean annual temperature, with an apparent activation energy (*E*b_chla_) of 0.53 eV (95% CI: 0.39–0.68; *r* ^2^ = 0.52; Fig. 4a). Annual community secondary production was strongly associated with mean annual chlorophyll *a* biomass (*r* ^2^ = 0.69; Fig. 4b). On average, a 10% increase in chlorophyll *a* biomass corresponded with a *c*. 12% (95% CI: 8.8–15.9%) increase in annual secondary production (Fig. 4b; log‐log slope = 1.2; 95% CI: 0.9–1.6).

**Figure 5:**
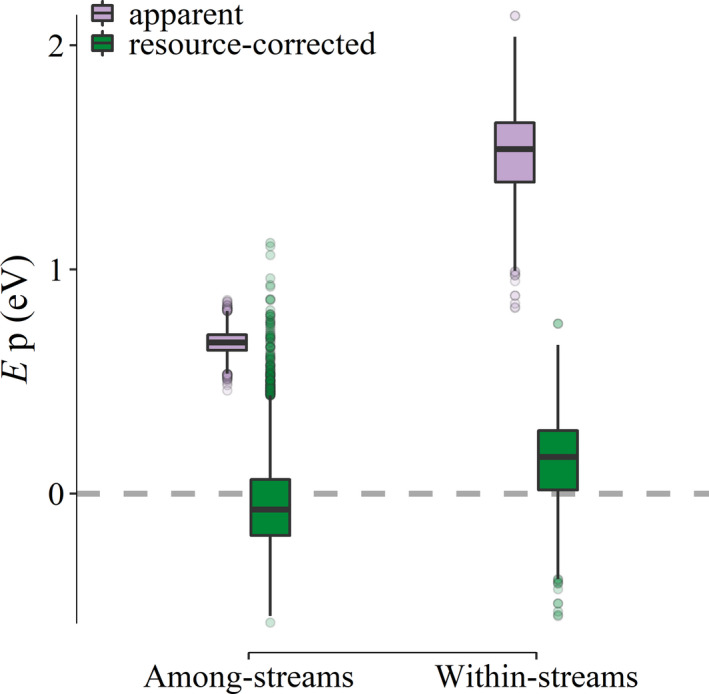
The temperature dependence of community secondary production (*E*p) before (‘apparent’; purple fill) and after (‘resource‐corrected’; green fill) correcting for resources. The apparent and corrected temperature dependence of secondary production among streams (mg AFDM m^−2^ y^−1^) were calculated as the temperature coefficient in the temperature‐only and additive temperature + resource linear models respectively. Within streams (mg AFDM m^−2^ d^−1^), the temperature fixed‐effect coefficient from the multivariate mixed‐effects model of secondary production (apparent temperature dependence) was corrected by subtracting the temperature coefficient of modelled gross primary production from two streams (see methods and Appendix S1). Box plots show the median (black line) and interquartile range (IQR; vertical box bounds). Whiskers represent ± 1.5*IQR and points beyond these bounds are printed individually.

Model selection consistently indicated that resource supply was a better predictor than temperature in explaining patterns of annual secondary production (i.e. 85% of bootstrap model selection events found chlorophyll *a* alone as the top model versus < 1% for the temperature‐only model; Table S2). Furthermore, the chlorophyll *a*‐only model was consistently better than more complex models (Table S2). After accounting for the influence of chlorophyll *a* biomass, the estimated temperature dependence of annual community secondary production overlapped zero (mean estimate: ‐0.05 eV; 95% CI: −0.40 to 0.37; Fig. 5), consistent with MTE predictions.

### Accounting for resource variation within streams

Within streams, temperature had no clear association with seasonal variation in chlorophyll *a* biomass (Fig. S5a). Similarly, chlorophyll *a* biomass was not consistently associated with seasonal variation in community secondary production (Fig. S5b). Patterns of community secondary production within streams were best explained by an additive mixed‐effects model that included chlorophyll *a* biomass, mean light availability and temperature as fixed effects, with a random effects structure allowing for stream‐specific intercepts (Table S3). This model estimated a relatively steep apparent temperature dependence of secondary production within streams (mean *E*p_within_ = 1.52 eV; 95% CI: 1.04–1.91; Appendix 2, Table S3), even after accounting for light and resource biomass. Using empirical measurements of GPP in two of our study streams, we estimated a within‐stream temperature dependence of GPP (*E*gpp_within_) of 1.37 eV (95% CI: 1.36–1.38; Table S4). After correcting for the variation in resource production (i.e. ‘resource‐corrected’ *E*p_within_ ‐ *E*gpp_within_), secondary production had a temperature dependence that overlapped 0 eV (Fig. 5, mean 0.14 eV, 95% CI: −0.33 to 0.54), consistent with temperature independence of secondary production within streams.

## DISCUSSION

Since the development of MTE, the fundamental roles of temperature and body size have been elevated as key drivers of ecological pattern and process. Although the importance of resource supply (i.e. the *[R]* term) has been clearly acknowledged within the MTE framework (Brown *et al*., 2004; Sterner, 2004; Kaspari, 2012), its influence on the metabolism of individuals and the production of animal communities has rarely been addressed. Here, we tested a central prediction of the metabolic scaling framework—the temperature independence of secondary production—using a robust empirical assessment of stream animal communities. We show that while growth rates of individuals generally followed the theoretical effects of body size and temperature, the apparent temperature dependence of production at higher levels of organisation was governed by variation in resource supply. Once we accounted for the influence of resource supply (i.e. chlorophyll *a* biomass among streams or primary production within streams), we found no evidence of a systematic effect of temperature on community secondary production. Our results provide strong empirical support for the temperature *independence* of animal community production and highlight the need to better incorporate resource variation in metabolic scaling efforts.

The predicted temperature independence of secondary production is an extension of the energy‐equivalence concept for body size (Damuth, 1987; Allen *et al*., 2002), which posits that population energy use and production should not vary systematically among organisms of vastly different body sizes (Ernest *et al*., 2003). Temperature independence arises from a similar mechanism in that it predicts that energy use and production should not vary with temperature because of the counteracting effects of temperature on standing biomass and biomass turnover (Huryn and Benke, 2007; White *et al*., 2007; Cross *et al*., 2015). The utility of the energy‐equivalence concept has been recently questioned because it assumes that resources are equally proportioned among populations in a community (Isaac *et al*., 2013). However, increasing evidence largely refutes this assumption, including Isaac *et al*. (2011) and our results which show large variation in secondary production among stream populations (*c*. 7 orders of magnitude; Fig. S3). Nevertheless, at the community‐level we found qualitative support for the mechanisms of compensation that underlie body size‐ and temperature‐independence at the annual scale. Specifically, annual community biomass (*B*) and rates of biomass turnover (*P:B*) showed opposing patterns across the temperature gradient. These did not equally counterbalance, however, leading to a positive apparent temperature dependence of annual secondary production (0.67 eV, Fig. 1a). Again, after accounting for resource supply, we found that the temperature sensitivity of community production was no longer present. Clearly, any robust test of energy‐equivalence or temperature‐independence must incorporate aspects of resource supply to consumers.

Strong control of resource supply on the temperature dependence of animal production was further supported by patterns of community biomass within streams. Here, it is likely that seasonal covariation between light and temperature led to a steepening of the apparent temperature dependence of secondary production (Fig. 3a). When light availability and resource production were low, most populations maintained low total biomass (Fig. S4). Such reduced biomass during dark and cool winter months led to an apparent temperature dependence of community biomass that countered MTE predictions *within* streams (i.e. positive slope; Fig. 3a), despite *among‐*stream patterns that qualitatively followed predictions (i.e. negative slope; Fig. 1b). We suggest that the consistently positive relationship between biomass and temperature *within* streams reflects adaptive coupling between the metabolic demands of species and their resources—specifically, a match between the timing of population biomass accumulation and the predictable availability of food resources (Ross, 1963). Such seasonal coupling of consumer energy demand and basal resource production may be a common attribute of mid‐ to high‐latitude ecosystems, with and without strong covariance between temperature and light regimes (e.g. Junker and Cross, 2014; Huryn and Benstead, 2019).

Using empirical measurements of resource production in a subset of streams, we showed that basal resource production could account for the amplified temperature dependence (i.e. *E*p_within_: 0.8–4.1 eV; Table 1) of secondary production within streams. While our resource production estimates were derived from only two of our study streams, and thus cannot completely account for the wide variation in *E*p_within_ values, other results from our study system show similarly amplified estimates of primary production across time and space. For example the apparent temperature dependences of stream biofilm (Welter *et al*., 2015; Williamson *et al*., 2016) and whole‐ecosystem NPP have been estimated as high as 2.8 eV (0.05–5.1 eV), consistent with biomass accumulating differentially among seasons or among different streams (Hood *et al*., 2018). Such large apparent effects of temperature highlight the importance of interactions among temperature, biomass and nutrients (Cross *et al*., 2015) that can influence estimates of activation energy at the ecosystem level (Anderson‐Teixeira *et al*., 2008). Such effects may lead to complex, and often unexpected, responses of consumer communities to warming (O'Gorman *et al*., 2017).

The strong association between resource supply and community secondary production found in our study contrasts with recent results from another study, where Nelson and coauthors (2017b) found no observable change in community secondary production in response to a *c*. 3 °C whole‐stream warming experiment, despite a tripling of primary production (Hood *et al*., 2018). In this experiment, increased primary production was partially attributed to dramatic summer blooms of a single species of green algae, *Ulva* sp., which was not an important food resource for consumers (Nelson *et al*., 2020). In our study, the primary producer communities were dominated by more edible epilithic diatoms, green algae and some cyanobacteria (Gudmundsdottir *et al*., 2011; O'Gorman *et al*., 2012; Junker, 2019). This difference may explain why we found that consumer secondary production more closely followed variation in primary producer biomass and growth. In addition, our study was conducted in ecosystems that have been ‘thermally acclimated’ for decades, with ample time for the adjustment and response of consumer communities to their resources. Hence, the divergent relationships between resource supply and consumer production on short and long timescales may reflect transient vs. equilibrium food web dynamics in response to temperature (Shaver *et al*., 2000; O'Gorman *et al*., 2014).

There is a long history of research recognising temperature as a fundamental determinant of ecosystem patterns and processes (Arrhenius, 1889). Our study has shown that while temperature strongly influences many components of the ecosystem, including animal community biomass, biomass turnover rates and the timing and magnitude of basal resource supply, temperature has minimal direct influence on animal community production. Instead, the apparent temperature dependence of animal production was mediated by the influence of temperature on basal resource dynamics. As global temperature regimes change in response to anthropogenic activities, predicting the response of animal production will require a greater recognition of complex direct and indirect effects of temperature, and their relation to other environmental controls, on the provision of resources that support metazoan demands (Huryn *et al*., 2014; Huryn and Benstead, 2019).

## AUTHORSHIP

WFC, JPB, JMH and ADH conceived the study, GMG and JSO provided field support and local arrangements, all authors helped with study design and data collection, JRJ performed analyses and wrote the first draft of the manuscript and all authors provided input on further manuscript drafts.

### Peer Review

The peer review history for this article is available at https://publons.com/publon/10.1111/ele.13608.

### Open Research Badges

This article has earned Open Data badge. Data is available at (https://doi.org/10.5281/zenodo.3995134, https://github.com/jimjunker1/temperature_independence)

## Supporting information

Supplementary Material

## Data Availability

Code and data for reconstructing the results and figures are available at: https://github.com/jimjunker1/temperature_independence and Zenodo: https://doi.org/10.5281/zenodo.3995134
