# Supplementary material for: Resource supply governs the apparent temperature dependence of animal production in stream ecosystems
Source: Ecol Lett. 2020 Oct 1;23(12):1809–19. doi: 10.1111/ele.13608 (PMC7702057; doi:10.1111/ele.13608)
Supplement: Supplementary file 1 — Supplementary Material [file ELE-23-1809-s001.docx]

**Supporting Information, Appendix S1: Additional tables and figures.**

Table S1. Physical and chemical characteristics of the study streams. Temperature represents the mean annual temperature in degrees Celsius, T_min_ and T_max_ represent annual minimum and maximum daily temperatures, and discharge represents the median annual values (L/s). Nutrient concentrations represent mean and one standard deviation from repeated water samples throughout the year (n = 5 – 12). Temp-Light correlation is Pearson’s correlation coefficients between daily light intensity and temperature.

| Stream | Temperature (℃) | T_min_ | T_max_ | Median Discharge (L/s) | DIN (NO_3_^-^ + NH_4_^+^mg L^-1^) | SRP (mg L^-1^) | DIN:SRP (molar) | Temp–Light correlation (Pearson’s *r*) |
| --- | --- | --- | --- | --- | --- | --- | --- | --- |
| hver | 27.2 | 9.3 | 34.8 | 21.0 | 0.007 ± 0.68 | 0.022 ± 0.004 | 0.7 | 0.67 |
| st6 | 17.6 | 12.0 | 24.6 | 13.7 | 0.026 ± 1.19 | 0.029 ± 0.002 | 2.0 | -0.09 |
| st9 | 11.2 | 5.0 | 15.0 | 3.0 | 0.003 ± 1.65 | 0.033 ± 0.003 | 0.2 | 0.48 |
| st7 | 5.8 | 1.7 | 9.5 | 3.9 | 0.027 ± 0.011 | 0.033 ± 0.002 | 1.8 | 0.41 |
| oh2 | 5.5 | 1.0 | 12.0 | 12.5 | 0.008 ± 0.002 | 0.050 ± 0.004 | 0.4 | 0.57 |
| st14 | 5.0 | 0.02 | 12.0 | 12.5 | 0.025 ± 0.88 | 0.013 ± 0.002 | 4.4 | 0.47 |

Table S2. Occurrence of top models describing annual community secondary production among streams selected from 1,000 bootstrapped events. The top model was determined as the model with the lowest AICc score from model subsets for each resampling.

| Model | n | Percentage (%) |
| --- | --- | --- |
| Chlorophyll *a*-only model | 852 | 85.2 |
| Full interactive model | 112 | 11.2 |
| Full additive model | 27 | 2.7 |
| Temperature-only model | 9 | 0.9 |

Table S3. Model coefficients from the top mixed-effects model describing seasonal community secondary production (mg AFDM m^-2^ d^-1^). Chlorophyll *a* represents biomass measured in each stream and sampling interval (mg m^-2^), ‘light’ represents the log_e_-transformed mean daily light intensity over a given interval (lux d^-1^), ‘Boltzmann-temperature’ represents the mean interval standardized Boltzmann-temperature (1/*kT_[15]_* – 1*/kT*). The apparent temperature dependence of within-stream secondary production (*E*p_within_) is calculated as the temperature coefficient. Pseudo-*r*^2^ values were estimated for fixed-effects only (marginal) and the full model with random stream intercepts (conditional).

| **Fixed effects term** | **Estimate** | **95% confidence bounds** | **Pseudo-*r^2^*** |
| --- | --- | --- | --- |
|  |  |  | marginal |
|  |  |  |  |
| log*_e_*(chlorophyll *a*) | 0.16 | 0.12 – 0.3 |  |
| log_e_(light) | 0.08 | 0.02 – 0.15 |  |
| Boltzmann-temperature (*E*p_within_) | 1.52 | 1.04 – 1.91 |  |
| -- | -- | -- | 0.57 |
| **Random effects: Stream intercepts** |  |  | conditional |
| hver | 0.18 | -0.68 – 0.88 |  |
| st6 | 2.74 | 1.51 – 3.70 |  |
| st9 | 1.23 | 0.31 – 2.15 |  |
| st7 | 0.60 | -0.002 – 1.13 |  |
| oh2 | 3.78 | 2.54 – 4.82 |  |
| st14 | 1.18 | 0.28 – 1.99 |  |
| -- | -- | -- | 0.88 |


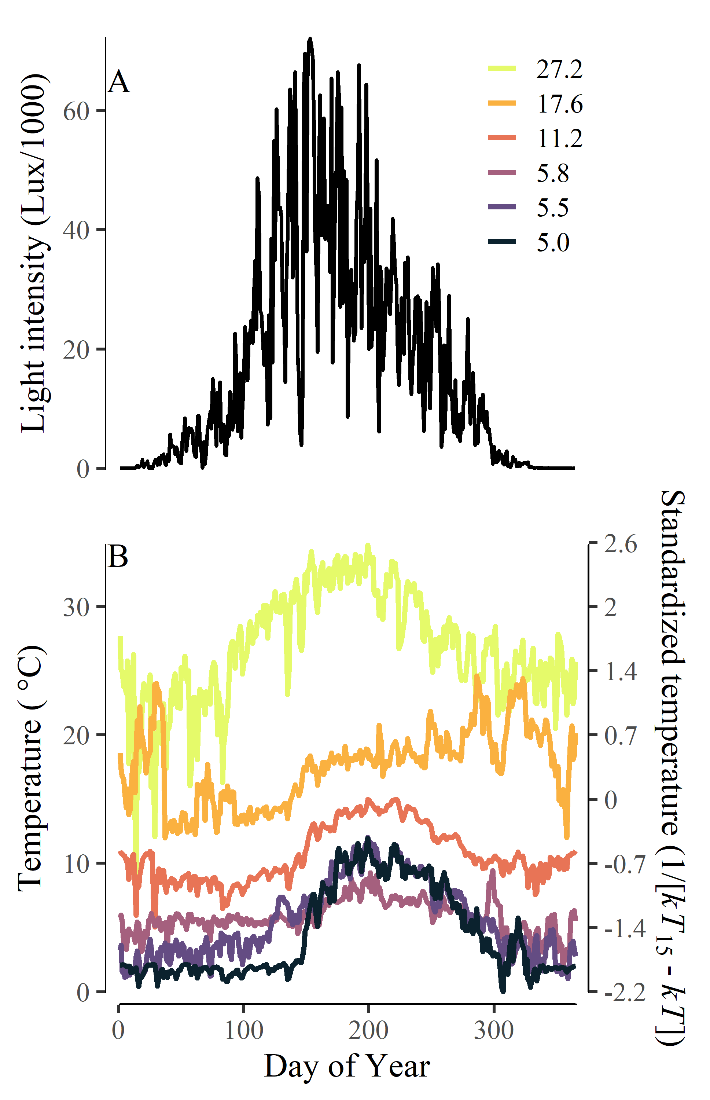
Figure S1. Annual patterns of (A) light intensity in the Hengill watershed and (B) mean daily temperature (°C) of study streams. Legend values show the mean annual temperature for each stream.


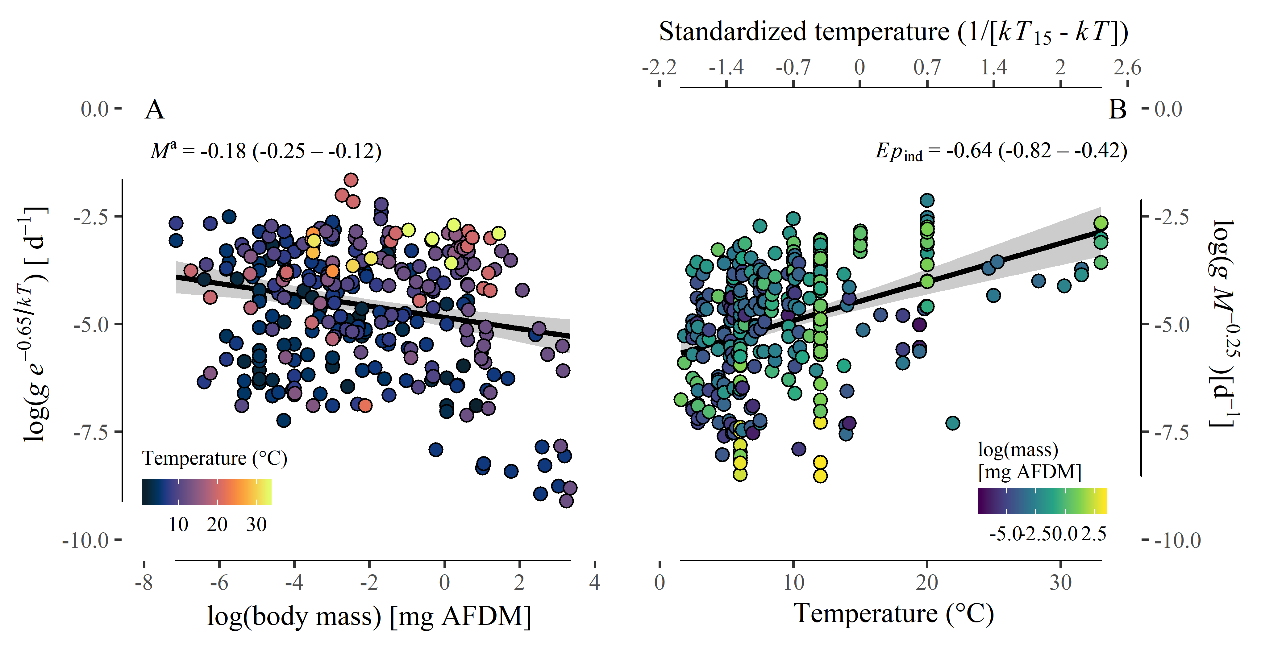


Figure S2. Patterns of individual instantaneous growth rates (*g*, d^-1^) and their relationship with (A) body size and (B) temperature. Growth estimates were calculated from *in situ* measurements and size-frequency shifts in identified cohorts. Each point represents the mean growth estimate for a unique stream-date-taxon combination. Mean and 95% confidence intervals of the multivariate linear regression fit show the body mass scaling (*M*^a^) and temperature dependence (*E*p_ind_) of individual growth rates. These estimates overlap with predictions of the metabolic theory of ecology (-0.25 and 0.65, respectively).


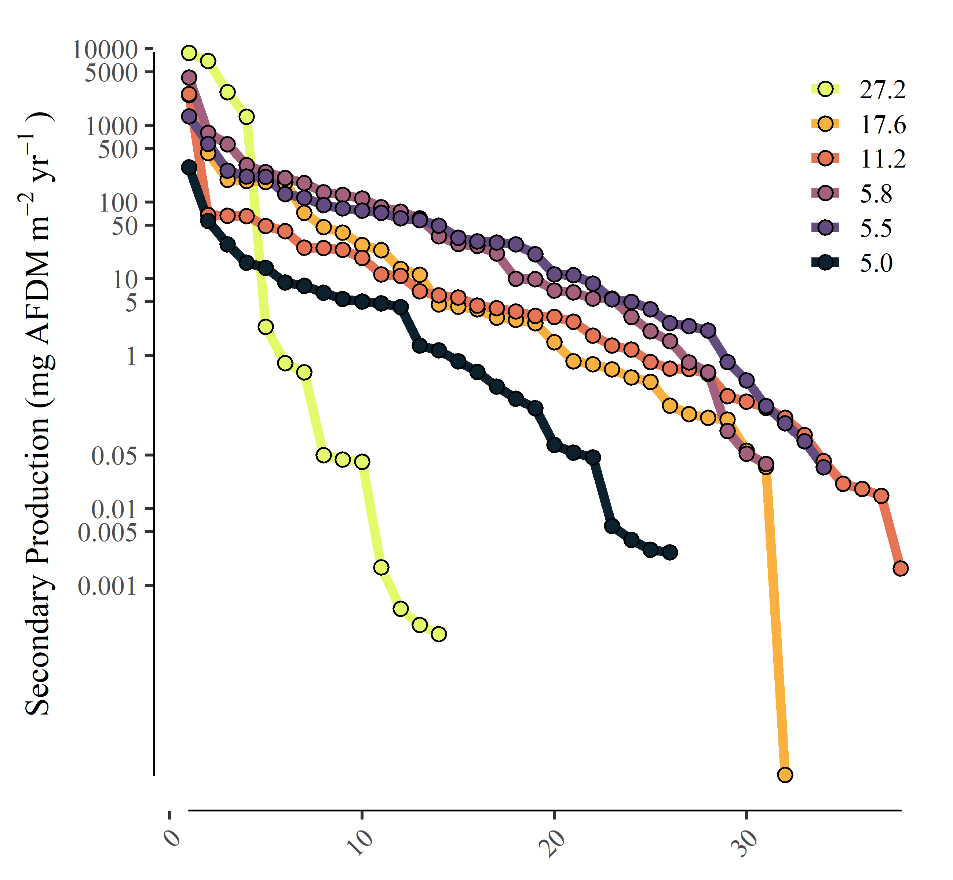


Figure S3. Ranked population-level annual secondary production (mg AFDM m^-2^ y^-1^) within each stream.


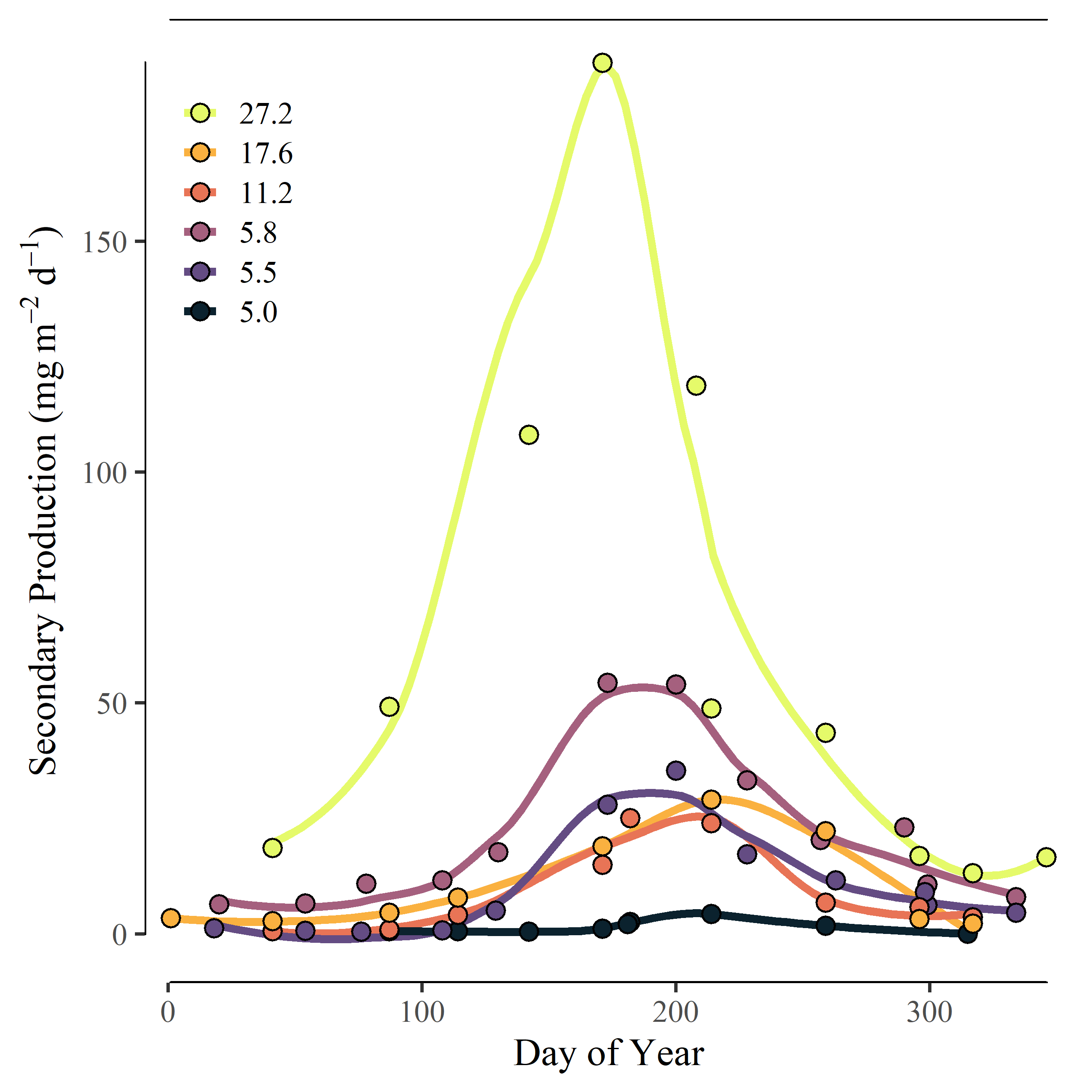


Figure S4. Community secondary production (mg AFDM m^-2^ d^-1^) throughout the year among study streams. Annual mean temperatures in ℃ are displayed in the figure legend. For visualization of general trends, locally weighted smoothing splines are shown (span = 0.6).


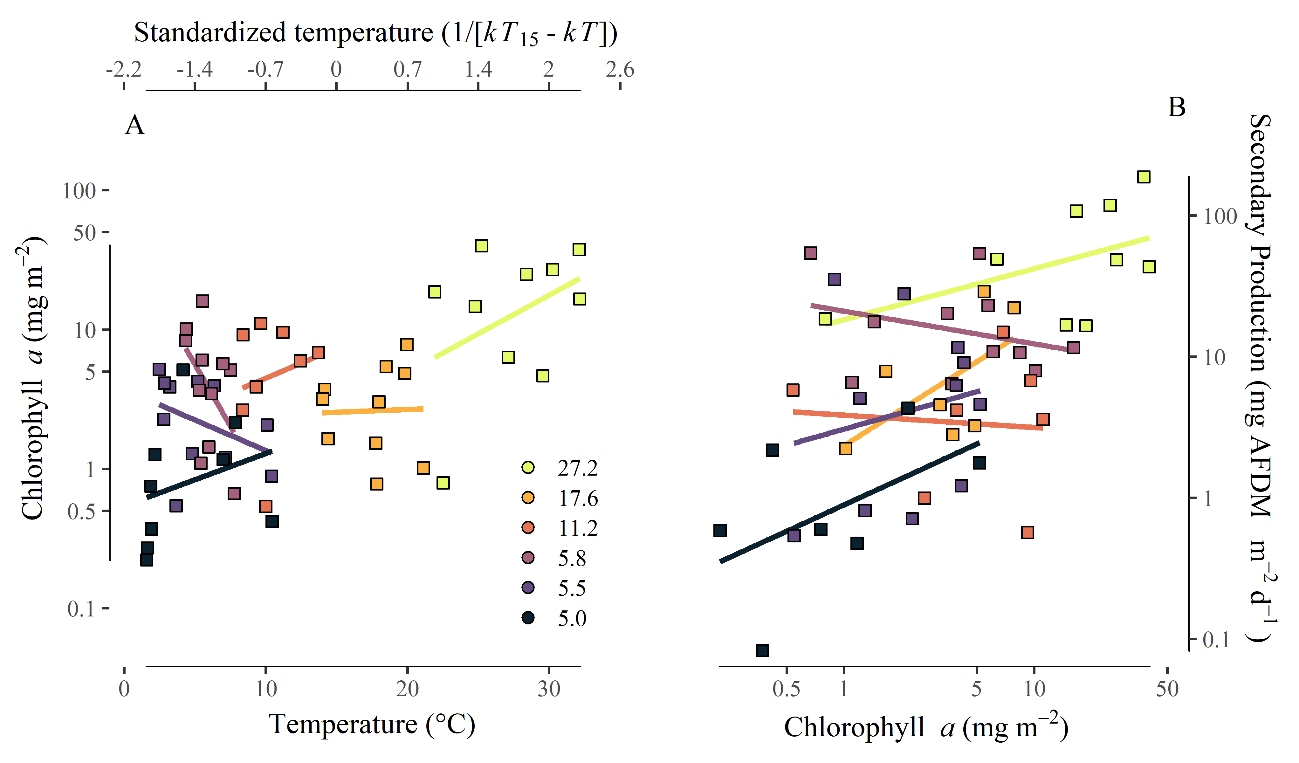


Figure S5. Relationships between temperature and chlorophyll *a* biomass (A) and chlorophyll *a* biomass and community secondary production (B) within and across streams. Solid lines represent the ordinary least squares fit to mean interval-level data within streams.

**Supporting Information, Appendix S2: Estimating the temperature dependence of resource supply within streams**

To account for variation in resource supply within streams, we must account for the effects of temperature on the *turnover* and *production* of resources. To accomplish this, we leveraged our previously published data from a subset of study streams (Hood et al. 2018; Figure S6) to develop an empirical model that predicts gross primary production (GPP) from light and temperature and chlorophyll *a* biomass. We used this model to estimate the temperature dependence of resource production, and to ‘resource-correct’ our estimates of the temperature dependence of secondary production within streams.

We fit a model for daily GPP (*GPP_within_*) using the same structure as the multivariate model for seasonal secondary production. Specifically, this model was a mixed-effects model with log*_e_*-transformed chlorophyll *a* biomass (*chla*), mean standardized Boltzmann temperature (*temperature*), and mean log_e_-transformed light availability (*light*) over a sampling interval as fixed-effects. Each stream was allowed a unique random intercept (ε^s^), leading to the full mixed model structure:

log_e_(*GPP_within_*) = log_e_(*chla*)+*temperature*+log_e_(*light*)+ε^s^

We estimated variability in model coefficients through a Monte Carlo approach that randomly permuted daily estimates of GPP. Posterior distributions of GPP were reconstructed (i.e. log-normal distribution fit to the 2.5^th^, 50^th^, and 97.5^th^ percentiles) to estimate the mean and standard deviation using the ‘get.lnorm.par()` function from the rriskDistributions package (Belgorodski *et al.* 2017). These distributions were then used to randomly generate 1,000 values of GPP on each day and construct 1,000 annual time series of GPP. These time series were summed over each sampling interval and fit using the model above. From each of these model fits we extracted the temperature coefficient as an estimate of the seasonal temperature dependence of GPP (*E*gpp_within_) accounting for both light and chlorophyll *a* biomass.

Modeled GPP was strongly associated with light availability and temperature (Figure S7A & B). Chlorophyll *a* biomass had a moderate, negative influence on GPP (Figure S7C). The full resource production model explained ~68% of the variation in daily GPP (Figure S7D) and the temperature dependence of GPP was 1.37 eV (95% CI: 1.36 –1.38; Table S4).

Table S4. Averaged mixed-effects model coefficients describing seasonal gross primary production (g C m^-2^ d^-1^). ‘light’ represents the log_e_-transformed mean daily light intensity over a given interval (lux d^-1^), ‘Boltzmann-temperature’ represents the mean interval standardized Boltzmann-temperature (1/*kT_[15]_* – 1*/kT*). Pseudo-*r*^2^ values were estimated for fixed-effects only (marginal) and the full model with random stream intercepts (conditional).

| **Fixed effects term** | **Estimate** | **95% confidence bounds** | **Pseudo-*r^2^*** |
| --- | --- | --- | --- |
|  |  |  | marginal |
|  |  |  |  |
| log*_e_*(chlorophyll *a*) | -0.147 | -0.149 – -0.145 |  |
| log_e_(light) | 0.468 | 0.465 – 0.471 |  |
| Boltzmann-temperature (*E*gpp_within_) | 1.37 | 1.36 – 1.38 |  |
| -- | -- | -- | 0.68 |
| **Random effects: Stream intercepts** |  |  | Conditional |
| st7 | -3.51 | -3.54 – -3.47 |  |
| oh2 | -3.51 | -3.54 – -3.47 |  |
| -- | -- | -- | 0.68 |


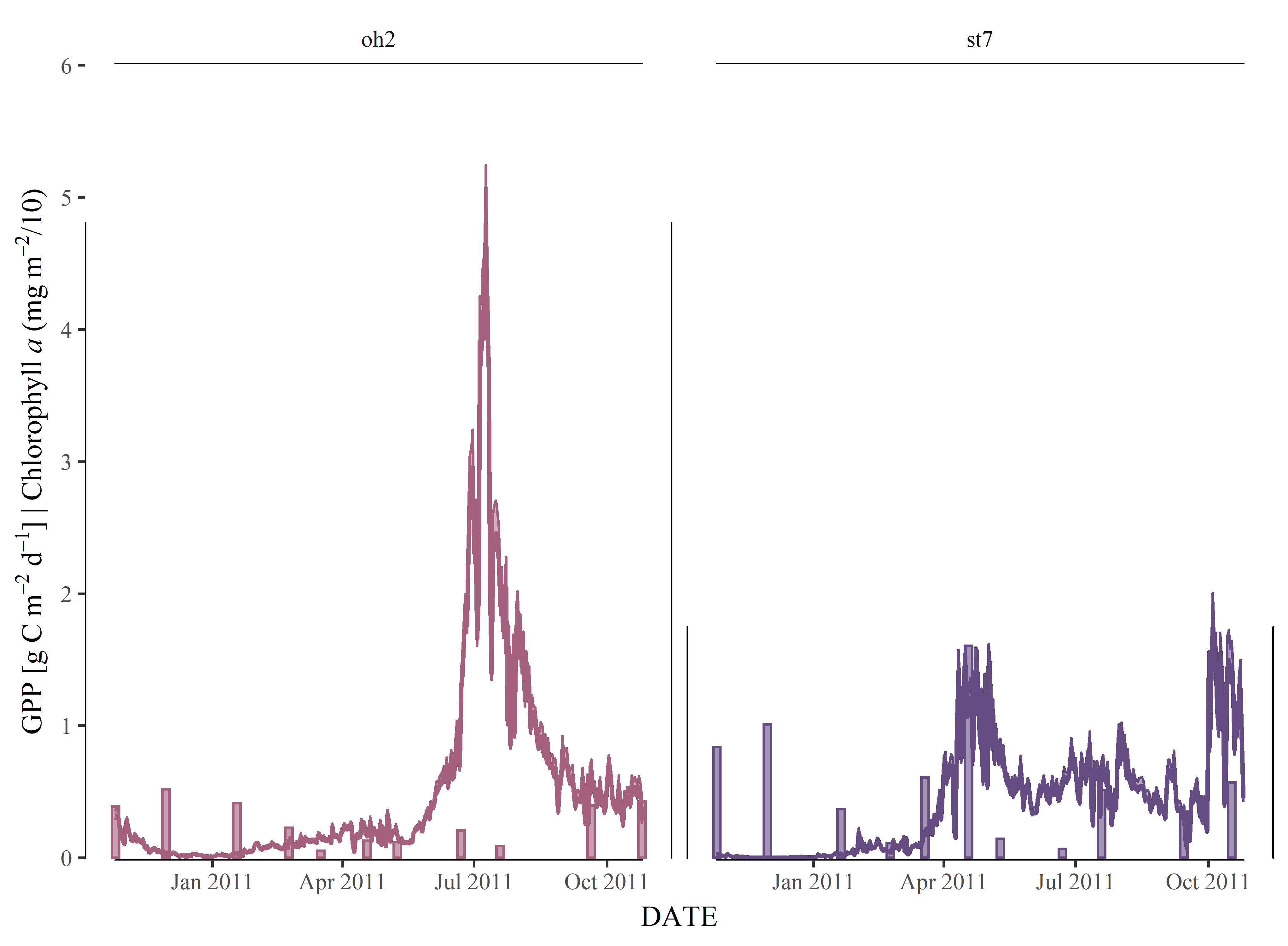


Figure S6. Patterns of daily gross primary production (GPP, g C m^-2^ d^-1^, solid lines) across the year and interval measurements of chlorophyll *a* biomass (vertical bars, mg m^-2^) in two study streams.

Figure S7. Bivariate relationships between mean daily gross primary production (GPP) and mean interval (A) light availability and (B) Boltzmann-standardized temperature and (C) chlorophyll *a* biomass in two study streams. The full model explained 68% of the variation in mean daily GPP throughout the year (D). Lines represent ordinary least squares fit to observed data. These data were originally presented by Hood and coauthors (2018) and are summarized here to estimate the within-stream temperature dependence of resource supply.


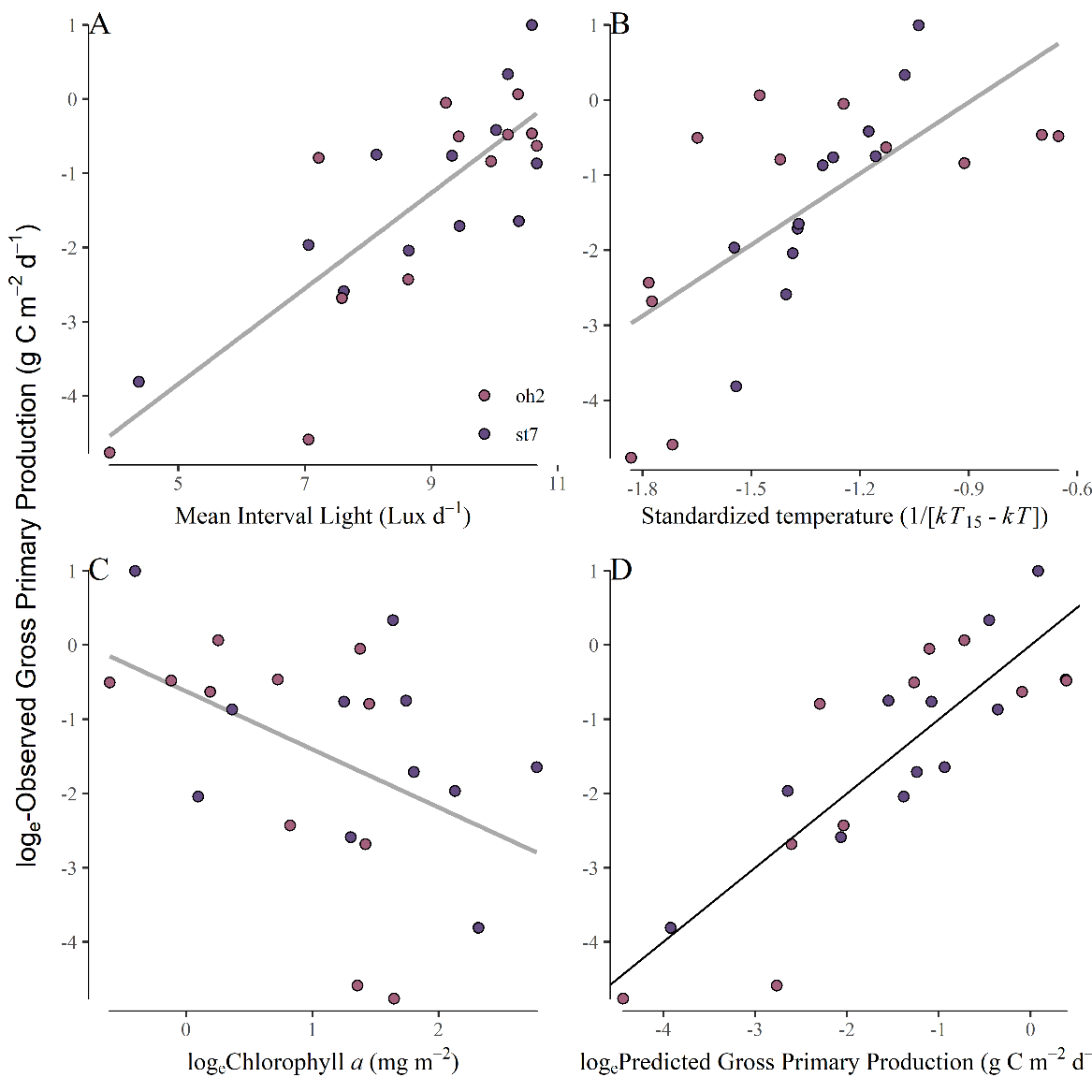


**Literature cited:**

Belgorodski, N., Greiner, M., Tolksdorf, K. & Schueller, K. (2017). rriskDistributions: Fitting Distributions to Given Data or Known Quantiles.

Hood, J.M., Benstead, J.P., Cross, W.F., Huryn, A.D., Johnson, P.W., Gislason, G.M. *et al.* (2018). Increased resource use efficiency amplifies positive response of aquatic primary production to experimental warming. *Global Change Biol.*, 24, 1069-1084.
